# A novel nomogram and a simple scoring system for urinary leakage after percutaneous nephrolithotomy

**DOI:** 10.1590/S1677-5538.IBJU.2022.0091

**Published:** 2022-05-18

**Authors:** Murat Sahan, Serkan Yarimoglu, Salih Polat, Bilal Nart, Omer Koras, Ibrahim Halil Bozkurt, Tansu Degirmenci

**Affiliations:** 1 HSU Izmir Bozyaka Training and Research Hospital Department of Urology Izmir Turkey Department of Urology, HSU Izmir Bozyaka Training and Research Hospital, Izmir, Turkey; 2 Amasya University Faculty of Medicine Department of Urology Amasya Turkey Department of Urology, Amasya University Faculty of Medicine, Amasya, Turkey; 3 Hatay University Faculty of Medicine Department of Urology Hatay Turkey Department of Urology, Hatay University Faculty of Medicine, Hatay, Turkey

**Keywords:** Kidney Calculi, Nephrolithotomy, Percutaneous, Urinary Incontinence, Stress

## Abstract

**Introduction:**

The present study aimed to investigate the factors of prolonged urinary leakage (PUL) after percutaneous nephrolithotomy (PCNL) and develop a new and simple scoring system to predict it.

**Patients and Methods:**

We retrospectively reviewed patients with renal stones who underwent PCNL at the University of Health Sciences Izmir Bozyaka Training and Research Hospital between April 2011 and January 2020. The patients were divided into two groups according to the presence of PUL, and their preoperative and perioperative data were compared. A multivariate regression analysis was applied to examine the relationship between perioperative descriptors and PUL, and a nomogram was developed using significant predictors. Then, the individual components of the nomogram were assigned points to form a scoring system.

**Results:**

There were 92 and 840 patients in the groups with and without PUL, respectively. The results of the univariate logistic regression analysis showed that hydronephrosis grade, parenchymal thickness, duration of nephroscopy, and duration of nephrostomy catheter were significantly associated with PUL. Subsequently, a multivariate regression analysis was carried out with these four factors as possible independent risk factors of PUL after PCNL. Based on the results of this analysis, a nomogram prediction model was developed with an area under the curve value of 0.811, which was consequently used to develop a new simple score system consisting of three characteristics: parenchymal thickness (1–5 points), duration of nephroscopy (1–3 points), and hydronephrosis grade (1–3 points).

**Conclusion:**

A novel scoring system is a useful tool for predicting PUL in patients who have undergone percutaneous nephrolithotomy.

## INTRODUCTION

Percutaneous nephrolithotomy (PCNL) is the standard treatment for renal stones of >2 cm (1). High stone-free rates reaching 96.1% can occur in PCNL (2). Despite the high efficacy of PCNL, complications of up to 20-83% are described in the literature. The most common of these complications are postoperative fever (4-32.1%), bleeding requiring transfusion (10.9-17.5%), and urine extravasation (7.2%) (3-5). However, when complications are classified according to the Clavien scoring system, the most common grade-3 complication is urine leakage persisting for >24 h (4%) treated by a double-J (DJ) ureteral stent (3).

After PCNL, a percutaneous nephrostomy (PCN) is placed in most cases for one to two days to provide both hemostasis and improvement in the access area (6). However, when the nephrostomy tube is removed at the end of this period, urine extravasation from the nephrostomy tract may sometimes continue. Nevertheless, a recent review by Xun Y et al. reported that patients who underwent tubeless PCNL, rather than standard PCNL, were associated with a lower risk of postoperative urine leakage (7). This is a disturbing event for both the patient and the physician. Although this returns to normal after the placement of a retrograde DJ stent, it requires re-anesthesia and an additional invasive procedure.

Many scoring systems have been developed to predict the results of PCNL (8-11). A previous meta-analysis also showed that complications could be predicted with scoring systems for PCNL (12). However, such scoring systems were not effective in showing prolonged urinary leakage (PUL). Although risk factors, such as hydronephrosis grade, duration of PCN catheter, type of dilator, PCN catheter diameter, renal parenchymal thickness in access line, residual stones, and mean stone burden have been identified for the development of PUL after PCNL, no scoring system is available to separately predict PUL (13, 14).

In this study, factors related to PUL were evaluated to predict which cases should receive a DJ catheter intraoperatively to shorten the length of hospital stay caused by PUL and decrease the exposure to additional anesthesia due to postoperative DJ catheter requirement, and a novel scoring system was developed to predict PUL after PCNL.

## PATIENTS AND METHODS

Patients who underwent PCNL were analyzed at the Izmir Bozyaka Training and Research Hospital between April 2011 and January 2020, retrospectively. The study was approved by Ethical Board (Meeting/Decision No.2021/145). Patients with chronic renal failure, ureteropelvic junction obstruction, concurrent ureteral stones, lower urinary system symptoms, residual stones causing obstruction, those requiring an intraoperative DJ stent implantation, and those that underwent tubeless PCNL were excluded from the study.

The patients who did not develop PUL were defined as Group 1, and those that developed PUL as Group 2. The patients who were immediately dry or experienced urinary leakage for less than 24h after nephrostomy removal were evaluated in Group 1. Retrograde DJ stents were placed in all patients with urinary leakage that lasted for >24h, and these patients constituted Group 2. Of the patients with >24 hours of urinary leakage or /and who were symptomatic, those with opaque stones were evaluated with ultrasonography and those with non-opaque stones were evaluated with non-contrast computed tomography (CT). The patients with residual stones causing obstruction were excluded from the study. The presence of urine leakage was determined by patient-reported wet dressings and/or hourly checks by the physician associate.

All the patients were preoperatively evaluated with a multi-slice plain CT. The degree of hydronephrosis was calculated according to the Society for Fetal Urology Hydronephrosis Grading System (15). We defined the renal parenchymal thickness as the access line distance from the renal capsule to the apex of the pyramid in the coronal plane CT images. The skin-to-parenchyma distance was defined based on the distance indicators on the needle, which was accessed using an 18-G initial puncture needle during the operation. Perioperative and postoperative data included operative time, duration of nephroscopy, duration of fluoroscopy, length of hospital stay, calyx of puncture, puncture site, access number, duration of PCN catheter, and presence of residual stones. The nephroscopy time was recorded from the access to the collecting system of the kidney to antegrade pyelography. The presence of residual stones was evaluated with fluoroscopy image and visual evaluation at the end of surgery. Sterile urine culture was detected in all patients before the operation.

The stone burden was calculated in square millimeters in all patients: length x width x ϖ x 0.25, where ϖ is a mathematical constantequalto 3.14 (16). In multiple intrarenal stones, the stone burden was calculated individually, and then the sum of all values were taken.

### Surgical procedure

After general anesthesia, a 5- or 6-F ureteral catheter was inserted into the collecting system of the kidney with stones and fixed to a Foley catheter. Then the patient was placed in the prone position, access was performed with an 18-G needle, and the tract was dilated with Amplatz dilatators to the 30 F caliber under fluoroscopy. A 26-F rigid nephroscope was used in the operation. Stones were fragmented with pneumatic lithotripter (Vibrolith; Elmed, Ankara, Turkey). At the end of the procedure, routinely, a 14-F nephrostomy tube was inserted. On the postoperative first day, the Foley catheter and the ureteral catheter were removed. The nephrostomy tube was removed on the postoperative first or second day in the absence of fever or significant hematuria after antegrade nephrostography showing ureteral drainage down to the bladder. After the nephrostomy tube was removed, the presence of a leak was defined by wet dressings either reported by the patient or determined by hourly checking in the ward by a resident. The decision for a dry patient was based on patient-reported comfort and doctor-determined dry dressing. Urinary leakage that persisted for more than 24 h was considered as PUL, and a retrograde DJ stent was placed in these patients. These procedures were routinely applied to all patients. Success was defined as the presence of asymptomatic residual stones of less than 4 mm or no evidence of stones on the postoperative first-month CT.

### Statistical Analysis

Categorical data were given as numbers and percentages. The conformance of continuous variables to normal distribution was evaluated using the Shapiro-Wilk test. Normally distributed variables were presented as mean and standard deviation, and those that did not show a normal distribution were presented as median and interquartile range. The independent-samples t-test was used to compare two independent normally distributed data, while the Mann-Whitney U test was used for the comparison of non-normally data. Pearson's chi-square and Fisher's exact tests were used in the comparison of categorical variables. Possible predictive variables associated with urine leakage were evaluated using multivariate logistic regression analysis, and the Backward elimination (Wald) method was used to create a model. The exclusion criterion for the model was set at p <0.1. A prognostic nomogram was constructed using the regression coefficients of independent predictive variables.

The predictive ability of the nomogram and scoring system was evaluated with the receiver operating characteristic (ROC) analysis. The nomogram was validated using the Bootstrap method (1,000 resamples). The scoring system was developed based on the score weights of the variables in the nomogram. The internal validation of the new scoring system was performed by calculating the score for each patient. Prediction ability was evaluated with the ROC analysis, and sensitivity and specificity values were calculated by determining the optimal cut-off value based on the Youden index. SPSS software (version 23.0; IBM Corporation, Armonk, NY, USA) was used for statistical analysis, and R-project statistical software and the ‘rms’ package included in this software were used for the nomogram.

## RESULTS

There were 932 patients in the study. There were 840 and 92 patients in Group 1 and Group 2, respectively. PUL was detected in 9.9% of the patients. The median age of the patients was 48 (38-57) years, and the median BMI was 26.1 (23.0-29.3). There was a history of extracorporeal shockwave lithotripsy (ESWL) in 169 (18.1) patients, history of ipsilateral surgery in 279 (29.9), and metabolic syndrome in 71 (7.6). The relationship between the demographic and preoperative characteristics of the patients and PUL are presented in Table-1. The two groups were similar in terms of renal pelvis anteroposterior diameter, stone location, stone density, and stone burden (p>0.005). The presence of hydronephrosis, a high hydronephrosis grade, and a decreased renal parenchyma thickness were found to be associated with PUL (p=0.014, p<0.001, and p<0.001, respectively). Residual Stone rates were similar between the groups (p=0,210). The relationship between the perioperative and postoperative outcomes of the patients and PUL is given in Table-1. Duration of nephroscopy, length of hospital stay, and length of PCN catheterization were significantly longer in Group 2 (p<0.001, p<0.001, and p=0.002, respectively).

**Table 1:** Comparison of demographic and preoperative characteristics of the patients according to the presence of urinary leakage.

Variables		Urinary Leakage		*p* value
		Absent (n=840)	Present (n=92)	
Age (years)		48 (38-57)	50.5 (37-60)	0.327
**Gender, n (%)**				0.273*
	Female	272 (32.4)	35 (38.0)	
	Male	568 (67.6)	57 (62.0)	
**Laterality, n (%)**				0.337*
	Right	394 (46.9)	48 (52.2)	
	Left	446 (53.1)	44 (47.8)	
BMI, kg/m^2^, median (25^th^-75^th^ percentile)		26.1 (22.9-29.4)	26.7 (24.2-29.4)	0.111
Metabolic syndrome, n (%)		61 (7.3)	10 (10.9)	0.216*
**Previous history of ESWL, n (%)**		155 (18.5)	14 (15.2)	0.534*
Ipsilateral surgery, n (%)		248 (29.5)	31 (33.7)	0.407*
Renal pelvis AP diameter		28.2 (19.7-60.0)	28.1 (19.3-47.2)	0.298
**Hydronephrosis, n (%)**				**0.014** *
	Absent	259 (30.8)	17 (18.5)	
	Present	581 (69.2)	75 (81.5)	
**Hydronephrosis, n (%)**				**<0.001** *
	Grade 0	259 (30.8)^a^	17 (18.5)^b^	
	Grade I	315 (37.5)^a^	21 (22.8)^b^	
	Grade II	154 (18.3)^a^	25 (27.2)^b^	
	Grade III	94 (11.2)^a^	24 (26.1)^b^	
	Grade IV	18 (2.1)^a^	5 (5.4)^a^	
**Stone location, n (%)**			
	Pelvis	220 (26.2)	30 (32.6)	0.164*
	Partial Staghorn	360 (42.8)	32 (34.8)	
	Staghorn	108 (12.8)	17 (18.5)	
	Multiple calyces	152 (18.1)	13 (14.1)	
Stone density, HU		1,100 (800-1,300)	1,100 (800-1,252)	0.828
Stone burden, mm^2^		314 (204-510)	282 (206-618)	0.831
Renal parenchymal thickness in access line, (mm)		15.4 (13.0-17.7)	11.8 (9.3-14.0)	**<0.001**
Skin-to-parenchyma distance, (mm)		80.0 (65-95)	79.2 (65-97.4)	0.942
**Calyx of puncture, n (%)**				0.210*
	Upper	34 (4.0)	8 (8.7)	
	Middle	285 (33.9)	29 (31.5)	
	Lower	468 (55.7)	48 (52.2)	
	Multiple	53 (6.3)	7 (7.6)	
**Puncture site, n (%)**				0.918*
	Supracostal	288 (34.3)	33 (35.9)	
	Subcostal	519 (61.8)	56 (60.9)	
	Multiple	33 (3.9)	3 (3.3)	
**Number of access, n (%)**				0.264*
	1	761 (90.6)	80 (86.9)	
	≥2	79 (9.4)	12 (13.0)	
Duration of operation, min.		90 (70-120)	100 (71.25-120)	0.850
Duration of nephroscopy, min.		40 (30-50)	50 (40-70)	**<0.001**
Duration of fluoroscopy, sec.		66 (42-102)	63 (46-97)	0.944
Length of hospital stay, days		3 (2-4)	4 (3-6)	**<0.001**
Duration of PCN catheter, days		2 (2)	2 (2-3)	**0.002**
Blood transfusion requirement, n (%)		70 (8.3)	6 (6.5)	0.547*
Residual stone, n (%)		216 (25.7)	34 (37.0)	0. 210*

**BMI**: Body massindex, **AP**: Anterior-posterior;

* Pearson'schi-square test

a, b = No significant difference between the same superscripts.

### Nomogram and simple scoring system development

**Figure 1:**
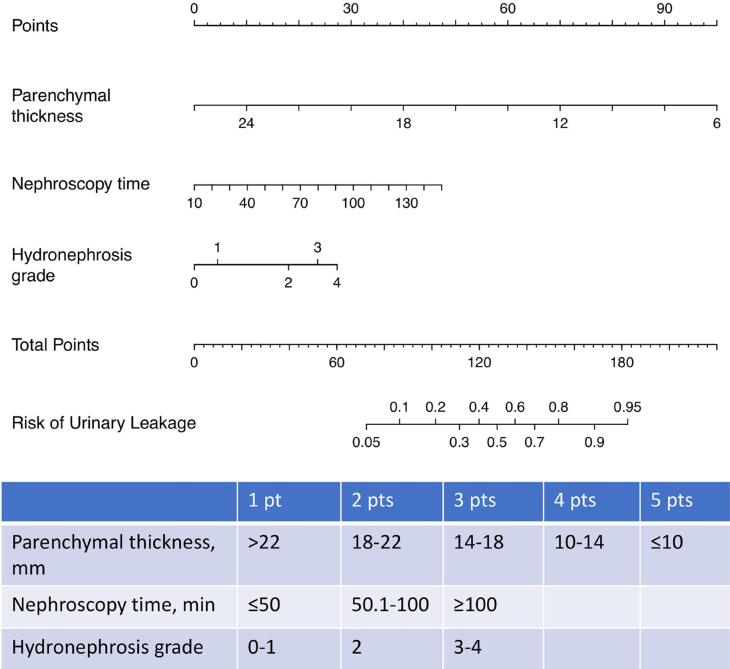
Nomogram and scoring system predicting urinary leakage after PCNL. The scoring system is based on radiological (parenchymal thickness, hydronephrosis grade) and surgical parameters (nephroscopy time). Parenchymal thickness (1–5 points), nephroscopy time (1–3 points), and hydronephrosis grade (1–3 points) are summed to provide a total score ranging from 3 to 11 points.

**Figure 2:**
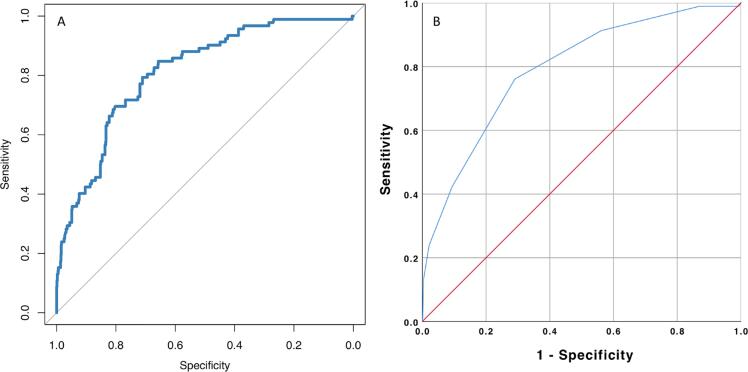
Receiving operator characteristic curve for predicting urinary leakage based on the nomogram and the scoring system. (a) The area under the curve (AUC) value of the nomogram was 0.811 (95% CI: 0.767-0.855) with an optimal cut-off value of 14.96%, at which it had a sensitivity of 77.2% and specificity of 74.2%. (b) The AUC value of the scoring system was 0.793 (0.745-0.841) with an optimal cut-off value of 6.5, at which it had 76.1% sensitivity and 71.0% specificity.

In the univariate analysis, hydronephrosis grade, parenchymal thickness, duration of nephroscopy, and duration of nephrostomy catheter were found to be associated with PUL (Table-1). The multivariate analysis conducted with these four variables revealed that hydronephrosis grade, parenchyma thickness, and duration of nephroscopy were independent risk factors for PUL (Table-2). Based on the results of the multiple regression analysis, a prognostic nomogram containing these three independent variables was developed (Figure-1). The area under the curve (AUC) value of the nomogram was 0.811 (95% CI: 0.767-0.855) with an optimal cut-off value of 14.96%, at which the model showed a sensitivity of 77.2% and specificity of 74.2% (Figure-2). The optimized corrected mean AUC value was determined as 0.800. Then, to be used in daily practice, a scoring system with a total score of 3 to 11 was created based on the effect sizes of a parenchymal thickness (1-5 points), duration of nephroscopy (1-3 points), and hydronephrosis grade (1-3 points) in the nomogram (Figure-1).

**Table 2:** Multivariate logistic regression analysis of possible factors in predicting urinary leakage.

		Multivariate analysis		Reduced multivariate analysis	
		OR 95% CI	p value	OR 95% CI	p value
**Hydronephrosis grade**					
	Grade 0	Ref		Ref	
	Grade I	1.317 (0.650-2.666)	0.445	1.270 (0.633-2.548)	0.502
	Grade II	2.658 (1.328-5.321)	**0.006**	2.624 (1.317-5.228)	**0.006**
	Grade III	3.548 (1.698-7.411)	**0.001**	3.536 (1.696-7.371)	**0.001**
	Grade IV	4.017 (1.199-13.457)	**0.024**	4.319 (1.270-14.689)	**0.019**
Parenchymal thickness		0.761 (0.710-0.816)	**<0.001**	0.765 (0.714-0.820)	**<0.001**
Duration of nephroscopy		1.017 (1.009-1.025)	**<0.001**	1.018 (1.010-1.026)	**<0.001**
Duration of PCN catheter		1.219 (0.936-1.588)	0.141		

**OR** = odds ratio

**Figure 3:**
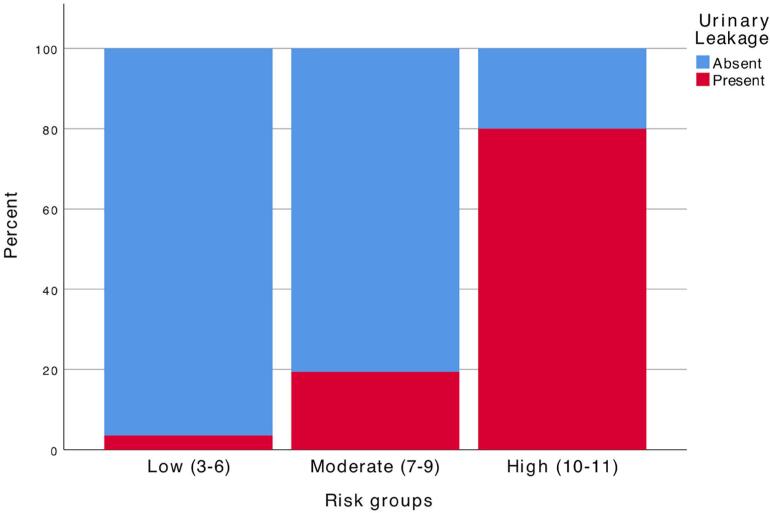
Stacked bar graph of the classification of urinary leakage risk as low, moderate, and high based on our score. Based on novel scores, the patients were divided into the risk groups of low (3-6), moderate (7-9), and high (10-11), which were found to have the PUL rates of 3.6%, 19.4%, and 80%, respectively.

The novel scoring system was applied to each patient and internal validation was performed. While the median score was 7 (7-8) in the patients with PUL, it was 6 (5-7) in those without PUL (p <0.001). The AUC value of the scoring system to predict PUL was 0.793 (0.745-0.841) (Figure-2). This value was comparable with the AUC value of the nomogram (0.800). The optimal cut-off value of the scoring system was 6.5, at which it had 76.1% sensitivity and specificity of 71.0% in predicting PUL. Based on novel scores, the patients were divided into the risk groups of low (3-6), moderate (7-9), and high (10-11) (Figure-3), which were found to have the PUL rates of 3.6%, 19.4%, and 80%, respectively.

## DISCUSSION

Despite its high stone-free rates and efficacy, large-scale complications can occur after PCNL (3, 4). The prolongation of urinary leakage from the nephrostomy tract after nephrostomy requires the insertion of a DJ catheter, which is classified as a grade 3 complication according to the Clavien scoring system (3). Residual stones migrate to the ureter and cause edema and blood clot obstruction, leading to PUL. The way to resolve this is the placement of a ureteral DJ catheter. In a study conducted by Binbay et al., 57 (4.3%) of 1,326 patients who underwent PCNL required a DJ catheter due to PUL, and this increased the length of hospital stay (13). Tefekli et al. supported the idea that PUL was the most common type of grade 3A complication that increased the duration of hospital stay (3). Similarly, in a recent study, the length of hospital stay was significantly longer in patients with PUL.

Although there are accepted scoring systems that predict success in renal stone treatment, these scoring are insufficient to predict the risk of complications. Thomas et al. proposed a scoring system (Guy's stone score, GSS) to preoperatively predict stone-free status by grading the complexity of PCNL. However, they found that although the score was associated with the stone-free rate, it was not associated with complications (8). Okhunov et al. indicated the necessity of additional studies to determine the role of the S.T.O.N.E. scoring system in predicting complications (9). In a single study of Ansari et al., GSS III and IV were reported to be associated with PUL (18). In the current study, we developed a new scoring system to prevent additional surgical interventions and reduce the length of hospital stay.

Previous studies have also evaluated the effect of hydronephrosis on urinary leakage from the nephrostomy tract after PCNL and shown that both the presence and degree of hydronephrosis are significantly associated with urinary leakage. Dirim et al. reported that the presence of hydronephrosis increased the incidence of urinary leakage, and a one-unit increase in the degree of hydronephrosis caused urinary leakage at the access site to prolong three times (14). The degree of hydronephrosis and the duration of urinary leakage have also been correlated in studies evaluating the relationship between the degree of renal hydronephrosis and long-term urinary leakage (13,17,18). Similarly, in our study, we showed that as the degree of hydronephrosis increased, the rate of PUL increased. It was also previously stated that a decreased parenchymal thickness caused less blood loss during PCNL (19). This may cause PUL due to delayed healing resulting from decreased blood supply to this area and the loss of the compressive properties of the thin parenchyma. Uyeturk et al. reported that the renal parenchymal thickness in the access line showed a more significant correlation with the duration of urinary leakage compared to the degree of hydronephrosis (20), which was also supported by Ansari et al. (18). In our study, we showed that renal parenchymal thickness in the access line was a factor predicting PUL.

Since residual stones may cause urinary obstruction after PCNL, operation success has been shown to be the strongest predictor of DJ catheter placement after PCNL due to PUL (21). Recent studies have stated that stone burden was associated with stone-free rates. So, increasing the stone burden affects the success rates negatively (22). Considering this information, it has been shown that both increased stone size and the presence of complex stones can predict the development of PUL after PCNL (13). Thomas et al. showed that most of the patients with GSS 3 and 4 required a second-look procedure due to multiple punctures and residual stones, and these patients developed more complications (8). Ansari et al. demonstrated that PUL was associated with GSS 3 and 4, multiple access attempts, and the presence of residual stones, but not with the stone burden (18). Dirim et al. found no significant relationship between stone burden and access number and urinary leakage (14). In our study, a significant relationship was not observed between PUL and the presence of residual stones, in addition, this complication was not related to access number and stone burden. In previous studies, it was not clearly stated whether residual stones caused obstruction (13, 18). Gucuk et al. reported that routine flexible nephroscopy during percutaneous nephrolithotomy was associated with a higher stone-free rate (23). However, in present study, flexible nephroscopy was not performed in any of the patients at the end of the procedure, so we could not evaluate its effectiveness. The feature that makes our study different from other studies is that patients with residual stones causing obstruction were not included in our sample. Although a previous study reported in a significant relationship between the skin-stone distance and PUL, the same authors did not reveal a similar relationship between this complication and the skin-calyx distance, which is a confusing finding (18). In our study, we found that skin-parenchyma distance was not associated with PUL.

Recently, the routine placement of a nephrostomy tube after an uncomplicated PCNL and complete stone cleaning have been questioned, except in required cases, such as those with residual stones, the possibility of a second-look procedure, significant intraoperative blood loss, and urine extravasation. It has been suggested that nephrostomy tubes cause postoperative discomfort and morbidities, such as urinary leakage and bleeding (24, 25). Therefore, there are studies supporting tubeless PCNL in the literature (25-27). In a study examining DJ catheter requirement due to urinary leakage after PCNL, Binbay et al. recommended using the tubeless approach in the treatment of small renal stones that are not complex (13). In another study conducted by Dirim et al., the prolongation of urinary leakage was determined to be in parallel with the time elapsed until the removal of the nephrostomy tube (14). In our study, a significant relationship was detected between the duration of nephrostomy tube use and PUL. To our knowledge, the effect of the duration of nephroscopy on urinary leakage from the nephrostomy tract after PCNL has not been previously evaluated. In the current study, the longer nephroscopy duration in the group with PUL can be explained by the greater stone burden and the higher number of staghorn stones in this group. We, therefore, determined that a longer duration of nephroscopy was a factor predicting PUL.

In previous studies, it has been shown that PCNL can be safely applied to patients with a history of ESWL or open nephrolithotomy with similar success and complication rates to those with no previous history of intervention (28, 29). Dirim et al. reported that a history of previous surgery or ESWL had no effect on urinary leakage following PCNL (14). Ansari et al. determined that a history of open surgery was not associated with PUL (18). Similarly, in our study, there was no relationship between previous surgery or ESWL history and PUL.

We thought that excessive bleeding requiring blood transfusion might influence the development of PUL with the mechanism of small blood clot formation causing pelvicalyceal system obstruction. Usually, small blood clots cannot be detected easily using the currently available radiological imaging tools. A previous study reported that there was no statistical correlation between bleeding and PUL development necessitating Double-J stent placement (13). Similarly, in our study, bleeding requiring blood transfusion was similar between the groups.

The novel scoring system created in our study had 76.1% sensitivity and 71.0% specificity in predicting PUL after PCNL. In the risk classification for a novel scoring system to be easily applicable in daily practice, the rate of PUL was found to be 80% in the patients in the high-risk group and 19.4% and 3.6% in the moderate- and low-risk groups, respectively. Considering this information, we recommend that intraoperative DJ stents should be placed in patients determined to have a high risk according to this classification.

Although the factors predicting PUL in our study were those that were previously proven to affect this complication and the results did not cause any confusion, our study has certain limitations. First, it had a retrospective and single-center design. Second, PCNL was performed on all patients with the same tract size, operation position, and lithotripsy technique, and the same-size nephrostomy tube and nephroscope were used. Third, since patients with intraoperative DJ stenting could not be included in the study, this may have possibly caused a bias in patient selection. Lastly, there was a relatively small number of patients with PUL. There is a need for prospective studies with larger series.

## CONCLUSION

The novel scoring system presented in this study is easy to use and repeatable. The efficacy of the factors predicting urinary leakage in the scoring system was demonstrated to be in agreement with the literature. In addition, this scoring system can be used as a predictive method to determine which patients should receive a DJ catheter intraoperatively to shorten the length of hospital stay by estimating the risk of urinary leakage and to decrease additional anesthesia exposure due to postoperative DJ catheter requirement.
